# A case of Trousseau syndrome: Screening, detection and complication

**DOI:** 10.1515/biol-2022-0824

**Published:** 2024-02-08

**Authors:** Hui Liu, Meng Jiang, Nan Wu, Qingxin Liu, Xueli Fan

**Affiliations:** Department of Neurology, Binzhou Medical University Hospital, Binzhou, China; Binzhou Medical University, Binzhou, China

**Keywords:** Trousseau syndrome, three territory signs, brain infarction, malignant tumors, cancer-related thrombosis, D-dimer levels

## Abstract

Trousseau syndrome (TS) is a malignant tumor-mediated complication of the hypercoagulable state with an unknown etiology. Laboratory testing results in patients with TS have indicated elevated D-dimer levels. The imaging analysis in patients who had undergone stroke has shown the presence of several cerebral infarction lesions in multiple regions. Since patients have had malignant tumors for a long time when they suffer from a secondary stroke, the optimum time for radical tumor treatment is usually missed. This study reports a case to improve the early screening and detection of TS and reduce the risk of recurrence of cerebral infarction.

## 1 Introduction

Trousseau syndrome (TS) is a migratory superficial thrombophlebitis condition initially described by Armand Trousseau in 1865. TS indicates a complication of the hypercoagulable state, which is caused by malignant tumors, such as pulmonary embolism, stroke, and lower limb intermuscular venous thrombosis, of which the malignancy-related stroke is the most risky complication [1]. Cancer-related thrombosis comprises the second leading cause of death in patients with cancer, with the first being cancer itself. Patients with cancer have a relatively higher risk of venous thromboembolism compared with patients without cancer. Thus, it is critical to assess the risk of thrombotic events and implement effective prevention and treatment in patients with cancer [2]. Patients often present with abnormally elevated D-dimer levels, and imaging findings have shown multiple regional cerebral infarction lesions. If the D-dimer levels are abnormally elevated in patients with unexplained stroke, and the imaging studies exhibit numerous cerebral infarctions in numerous regions, then these symptoms are highly suggestive of cryptogenic malignancies [3].

Previous studies have shown that lung cancer is the most common primary cancer in patients with cancer-related stroke, followed by hepatobiliary, gastrointestinal, and breast gynecological malignancies. Most patients exhibit adenocarcinoma as the primary histological subtype and systemic metastasis [4,5]. Here, the patient was diagnosed with pancreatic cancer. There is extensive literature and case discussions on pancreatic cancer-related cerebral infarction [6,7,8,9]. This study reports a case of TS caused by pancreatic cancer and characterized by several cerebral infarctions in multiple regions. The case study aims to improve clinical awareness of the diagnosis and treatment of TS, early detection, and intervention to reduce the risk of stroke recurrence.

## 2 Case description

A 78-year-old female was admitted to the Cardiology Department of the Binzhou Medical University Hospital on May 05, 2022. The patient had been experiencing chest tightness and fatigue for 3 days. There were no apparent causes of chest tightness and weakness, accompanied by abdominal distension, poor appetite, and dry stool. There was no chest pain, sweating, nausea, vomiting, cough, sputum, headache, dizziness, abdominal pain, diarrhea, and other discomfort. The chest tightness was usually experienced after activity, and the duration of symptoms varied. The patient had a history of hypertension for 25 years, with a systolic blood pressure of up to 170 mmHg, and was receiving oral treatment with “amlodipine besylate and atenolol.” After admission, the pulmonary artery CTA examination was performed, and the results showed multiple pulmonary embolisms and multiple low-density space-occupying lesions in the liver. An enhanced abdominal CT examination was recommended. Ultrasonography of deep veins in both lower extremities showed intermuscular venous thrombosis, which supported the diagnosis of pulmonary embolism. During the hospital stay, the patient was given anticoagulants, antihypertensives, acid inhibitory, and symptomatic treatment. The patient was discharged after the symptoms improved. The patient was prescribed the following medications: rivaroxaban tablets 15 mg qd, Rabeprazole sodium enteric-coated tablets 20 mg bid, and diltiazem hydrochloride tablets 30 mg tid.

On May 11, 2022, the patient was admitted to the Department of Neurology of Binzhou Medical University due to “numbness of the left limb for 4 days.” Approximately 4 days ago, the patient had developed left limb numbness and discomfort without any apparent cause, accompanied by left limb weakness, since the left hand could not hold objects, and the left leg was dragged while walking. The patient also experienced a drinking cough, swelling in the left lower limb, and blurred vision. There was no dysphagia, no headache, dizziness, no tinnitus, no disturbance of consciousness, no limb convulsions, no nausea, and vomiting, as well as no urinary and bowel incontinence. The symptoms persisted, and no special treatment was given outside the hospital.

The physical examination in the Neurology department revealed the following vitals: *T* – 36.6℃, *P* – 72 times/min, *R* – 18 times/min, BP – 136/84 mmHg. The patient had a clear mind, fluent speech, but a slow reaction. Bilateral pupils were equally large and round, with a diameter of approximately 3 mm, sensitive light reflex, free movement in both eyes, no staring, and nystagmus. The bilateral nasolabial exhibited grooved symmetry. The tongue extension was centered. The neck was soft, the right limb had a muscle strength of level 5, and the left limb had a muscle strength of level 4+; the muscle tension was normal, bilateral tendon reflex (++), and the left finger nose test was inaccurate. Bilateral heel knee and shin tests were not compatible. No apparent abnormalities were observed during the sensory examination, and the pathological signs were negative. The risk and prevention of VTE were assessed using the Padua scoring scale on admission. The score was 5 points, and the VTE risk level was high. At admission, the patient was diagnosed with the following: 1. acute cerebrovascular disease; 2. hypertension (grade 2, very high risk); 3. pulmonary embolism; 4. double calf intermuscular venous thrombosis; and 5. chronic gastritis.

After admission to the Department of Neurology, a brain CT was performed, and the results showed multiple lacunar infarctions, forming a partial softening lesion. Further, an MR Examination was recommended. The patient was administered rivaroxaban tablets, rosuvastatin calcium tablets, rabeprazole sodium enteric coated tablets, shuxuening injection, and ozagrel sodium injection. Subsequently, other relevant checks were done. The cervical vascular ultrasound showed multiple plaque formation in the right cervical artery and bilateral subclavian artery. The results of chest CT showed double lung fiber foci and double lung nodules; an annual review was recommended. A low-density shadow was observed in the liver and the tail of the pancreas, and further examination was recommended. The coagulation index was as follows: D-dimer 4.93 mg/L, fibrinogen content 1.1 g/L; platelet aggregation function: arachidonic acid, 3.53%; adenosine diphosphate, 35.29%; collagen, 36.59%.

**Figure 1:**
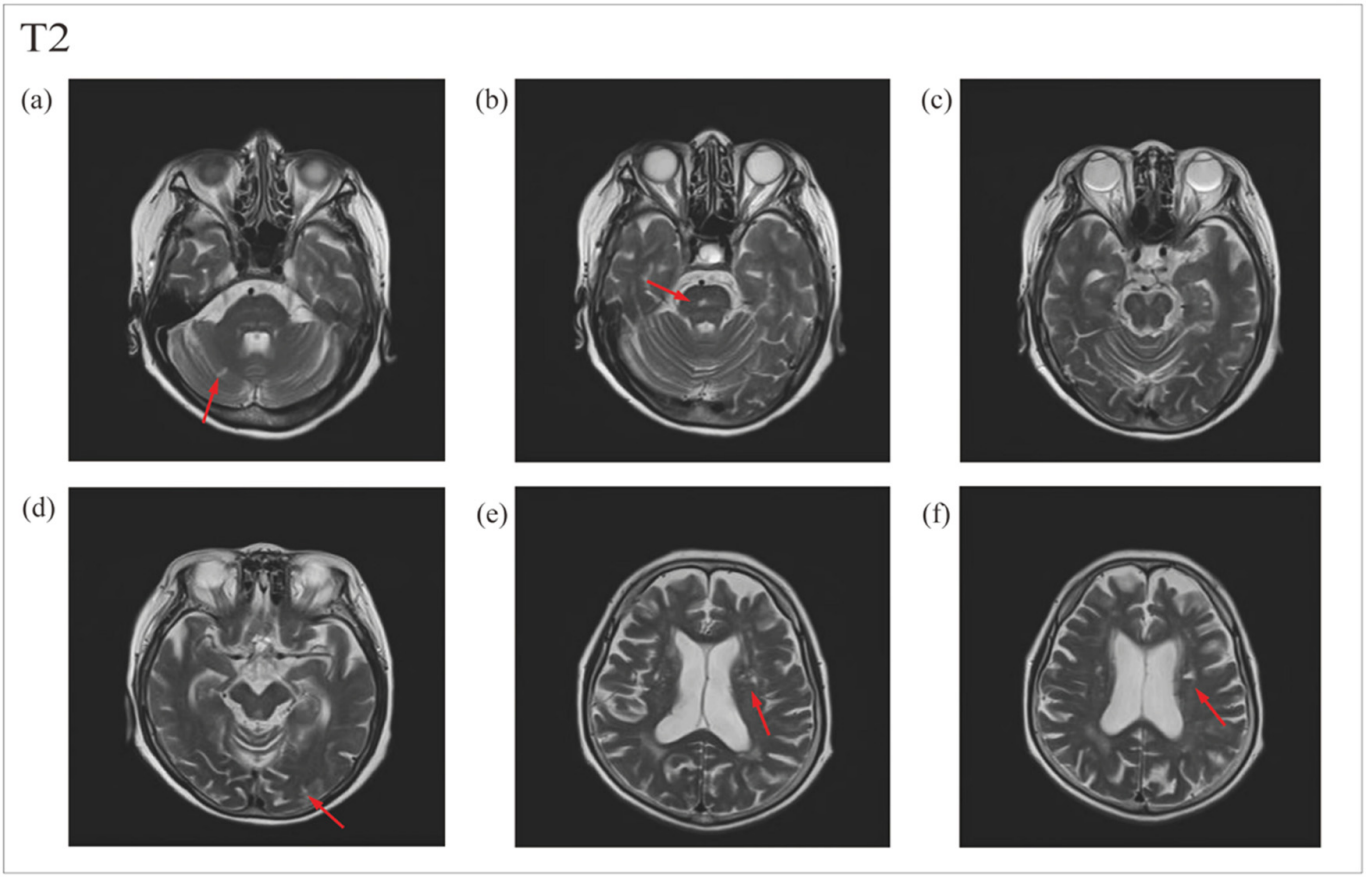
T2 sequence of cerebral MRI. (a–f) Multiple lacunar infarcts in cerebral cortex, subcortex and cerebellum.

**Figure 2:**
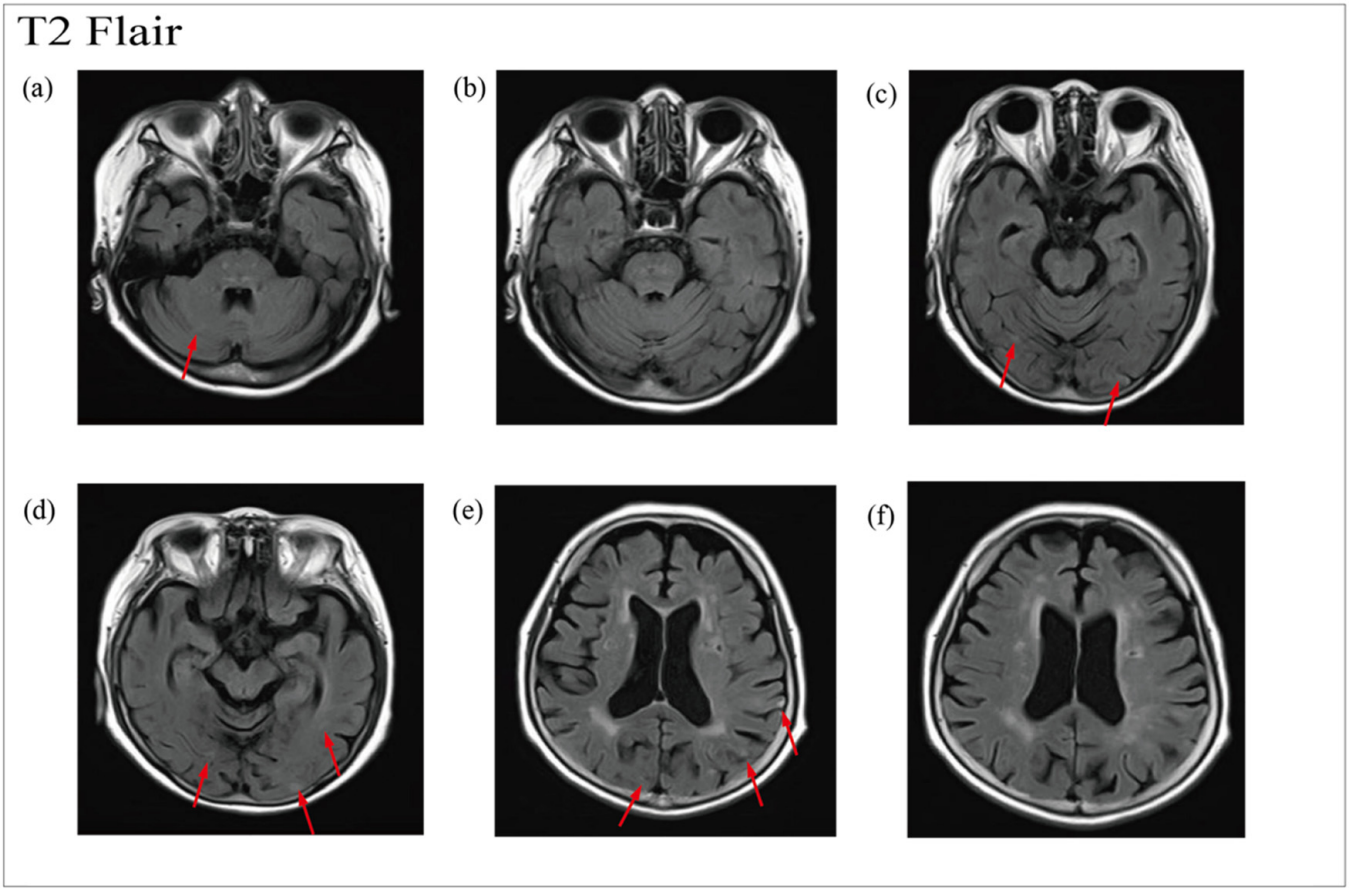
T2 Flair sequence of cerebral MRI. (a–f) Multiple lacunar infarcts in cerebral cortex, subcortex and cerebellum.

**Figure 3:**
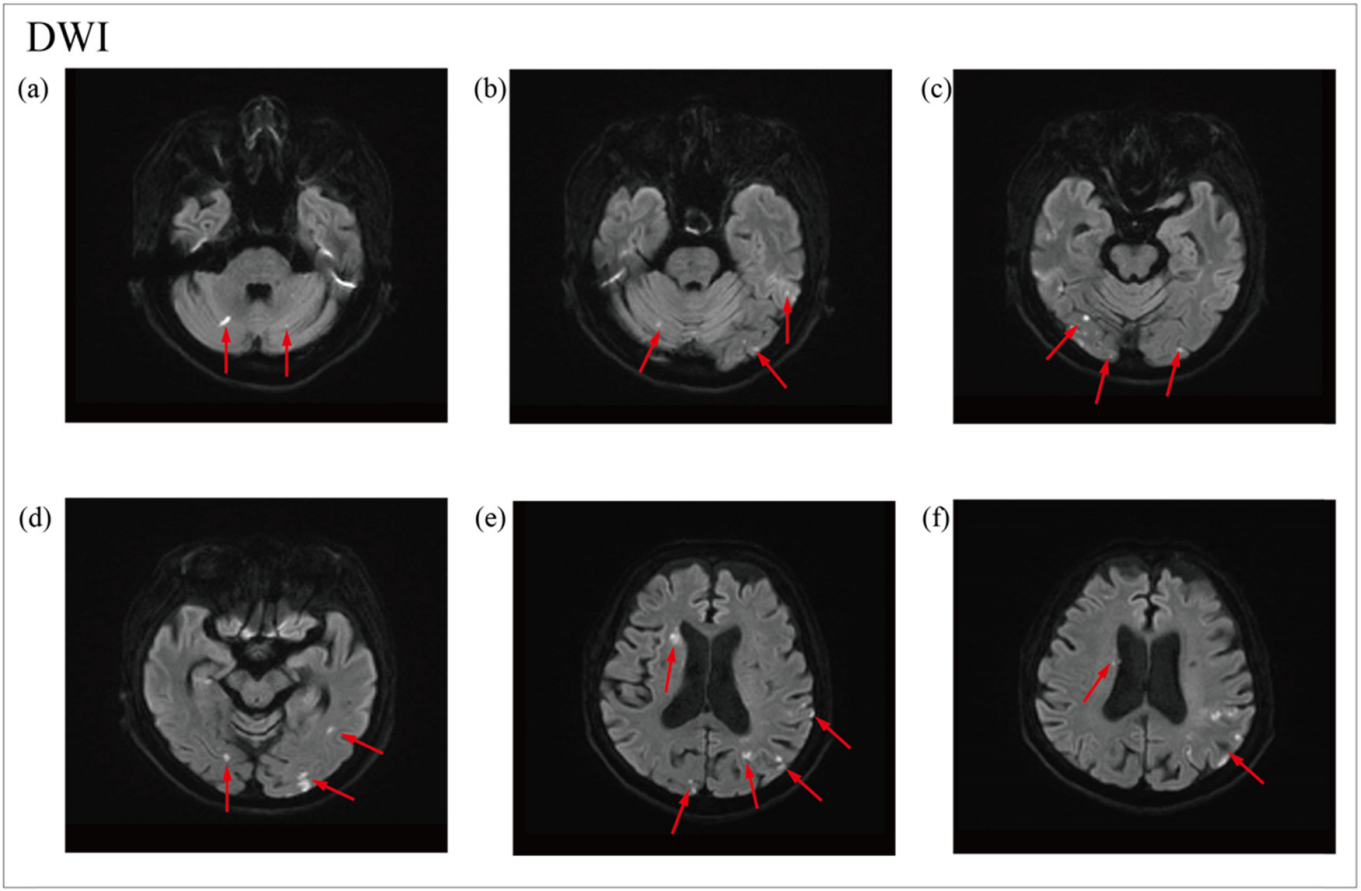
DWI sequence of cerebral MRI. (a–f) Multiple lesions of acute lacunar cerebral infarcion in cerebral cortex, subcortex and cerebellum.

**Figure 4:**
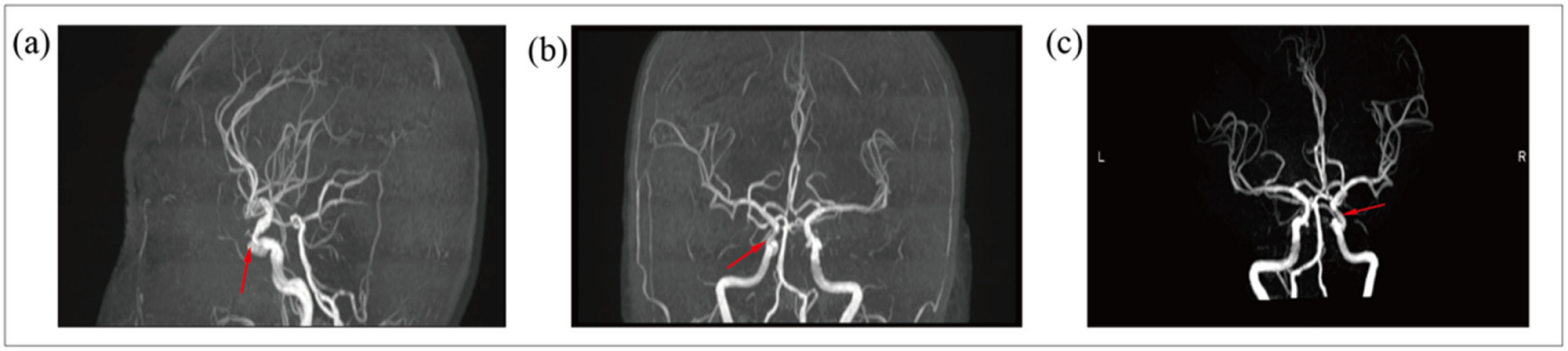
Cerebral MRA. (a–c) Cranial MRA suggests right internal carotid artery stenosis.

**Figure 5:**
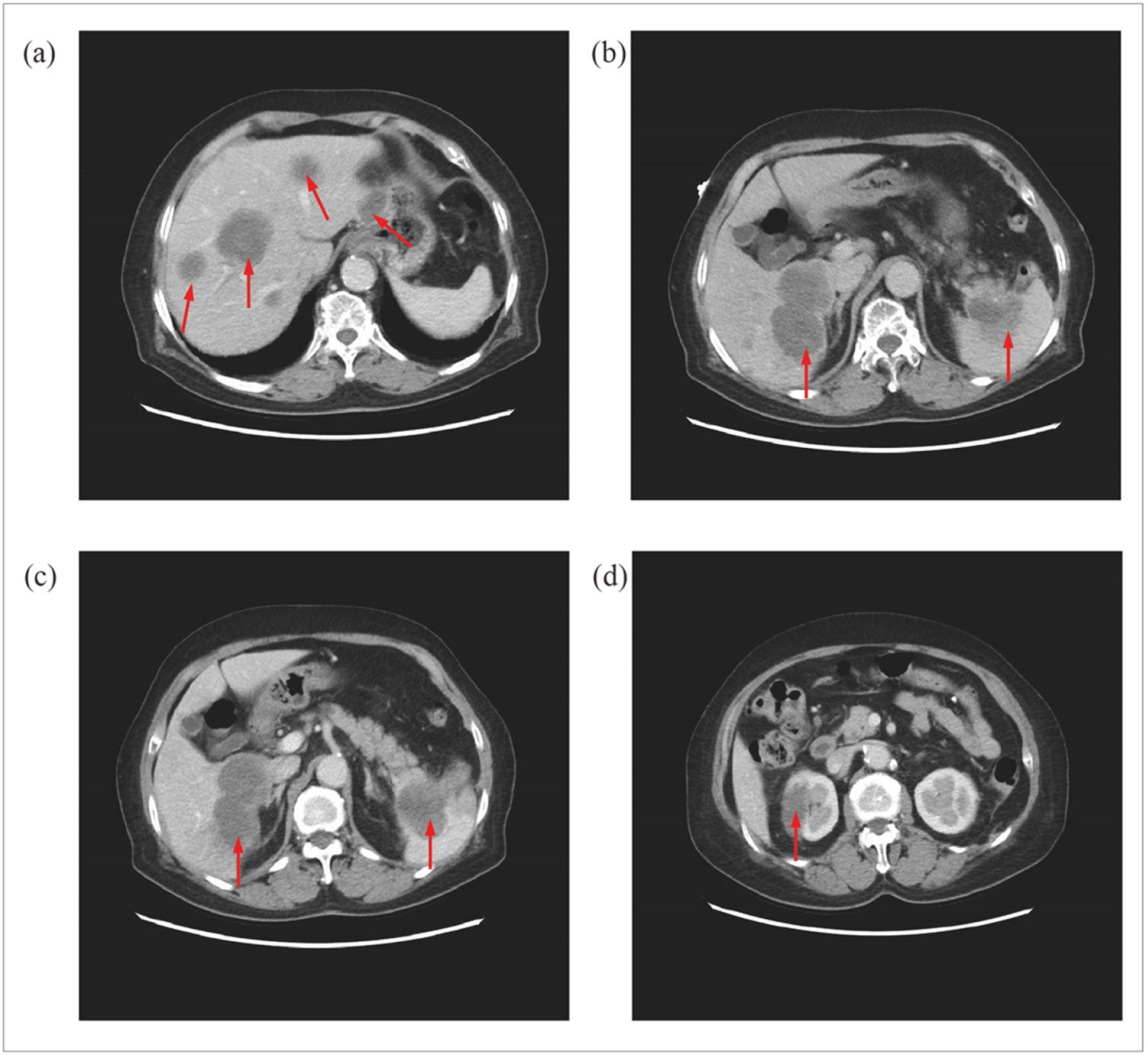
Abdominal enhanced CT. (a and b) Multiple cancer metastases in the liver and spleen. (c) Space occupying lesion of the pancreatic tail and the metastases in the liver. (d) Abnormal intensification in the right kidney.

**Figure 6:**
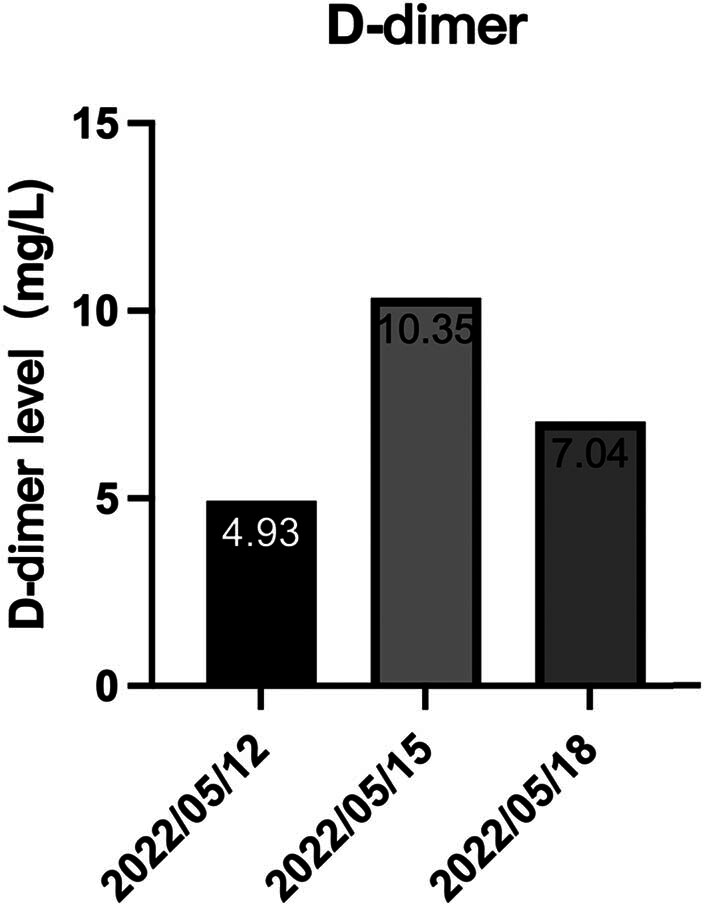
D-dimer level changes. The patient was admitted to the hospital on May 12, 2022 to check that the D-dimer level was higher than the normal level, and the patient was given low-molecular-weight heparin anticoagulation therapy on May 13, 2022. The D-dimer level was increased on May 15, 2022 and decreased on May 18, 2022. In the initial stage of low molecular weight heparin treatment, the level of D-dimer increased in the aggravated stage, and the level of D-dimer began to decline after low molecular weight heparin treatment for a period of time.

OnMay 12, 2022, a head MRA was done. The head MR Plain scan + DWI showed multiple acute lacunar infarctions (Figures 1–3). No significant abnormality was observed in the cerebral MRA. The right vertebral artery was more delicate compared with the contralateral (due to developmental considerations), and its initial area was less developed; these analyses were considered in combination with the results of other examinations (Figure 4). Further improvement of the five tumor examination results showed an increase in the tumor index (Table 1). Carcinoembryonic antigen, 244 ng/mL (normal range, 0–3.4 ng/mL); carbohydrate antigen (CA19-9), 378.40 U/mL (normal range, 0–27 U/mL); carbohydrate antigen (CA125) > 5000.00 U/mL (normal range, 0–35 U/mL); ferritin, 494.70 ng/mL (normal range, 13–150 ng/mL); and vitamin B12 determination, 1695.00 pg/mL (normal range, 190–940 pg/mL). The diagnosis of TS was considered. Anticoagulant therapy with low molecular weight heparin was administered on May 13, 2022. A re-examination of D-dimer results was done on May 15, 2022, which showed 10.35 mg/L. OnMay 15, 2022, the upper abdomen enhanced scan showed pancreatic tail occupancy, indicating pancreatic Ca and involvement of the spleen and adjacent blood vessels. Additionally, multiple abnormal enhanced foci in the liver and metastatic tumor were observed. Abnormal intensification in the right kidney, metastasis? An infarct? It was recommended to perform additional tests to check for double renal cysts and Coronary arteriosclerosis (Figure 5). A re-examination of D-dimer results on May 18, 2022 showed 7.04 mg/L. Based on the changing trend of D-dimer in patients (Figure 6), the disease was considered to be in the aggravated stage in the early stage of heparin treatment, and the levels of D-dimer increased. Treatment caused a gradual decrease in the levels of D-dimer, supporting the efficacy of the treatment.

**Table 1:** D-dimer and tumor five examination results

Test Name		Test Date	Test Result	Range of Normal Value
D-dimmer Level		May 12, 2022	4.93 mg/L	0–0.5 mg/L
		May 15, 2022	10.35 mg/L	
		May 18, 2022	7.04 mg/L	
Five items of cancer	Carcinoembryonic antigen	May 13, 2022	244 ng/mL	0–3.4 ng/mL
	Carbohydrate antigen (CA19-9)		378.40 U/mL	0–27 U/mL
	Carbohydrate antigen (CA125)		>5000.00 U/mL	0–35 U/mL
	Ferritin		494.70 ng/mL	13–150 ng/mL
	Vitamin B12 determination		1695.00 pg/mL	190–940 pg/mL

The patient had an advanced tumor without any indication of surgery. Stage IV pancreatic cancer was considered for consultation in the Department of Oncology, but the patient was in the acute stage of pulmonary embolism and cerebral infarction. Thus, anti-tumor therapy was recommended after the condition had stabilized. The patients were continuously administered anticoagulants, lipid-regulating plaques, medicines to improve circulation, and other symptomatic support treatments.

Patients with acute onset exhibit worsening of symptoms in a short time. The MRA examination of the patient’s brain showed multiple lacunar infarctions, and the results of blood clotting tests indicated hypercoagulability, tumor markers, and imaging examination showed advanced pancreatic cancer. Thus, the patient was diagnosed with TS. The left limb numbness of the patient had improved, and the patient and his family requested discharge. After discharge, the patient was administered 5000 IU of low molecular weight heparin calcium injection, q12h, Diltiazem hydrochloride tablet 30 mg tid, Rabeprazole sodium enteric-coated tablet 20 mg qd, and Mosapride citrate tablet 5 mg tid for continued drug treatment.


**Informed consent:** Informed consent has been obtained from all individuals included in this study.
**Ethical approval:** The research related to human use has been complied with all the relevant national regulations, institutional policies and in accordance with the tenets of the Helsinki Declaration, and has been approved by the authors’ institutional review board or equivalent committee.

## 3 Discussion

TS is usually defined as any type of unexplained thrombotic event occurring in the course of any type of malignant tumor. This definition mainly arises from the common hypercoagulable pathologic state exhibited by cancer patients. Consequently, the treatment of TS is primarily based on anticoagulant drugs [10].

Previous studies have suggested that substances in tumor cells, such as cysteine protease, tissue factors, and mucin sialic acid, possess pro-coagulant activities, which can result in the activation of coagulation factors X and XII [11,12,13]. Patients with tumors had elevated levels of tissue factor-positive microvesicles, which correlated with the level of D-dimers, suggesting that microvesicles containing tissue factors were involved in the activation of the coagulation pathway in patients with tumors [14,15,16]. The mechanism by which tumor cells promote coagulation function is still unclear, and further in-depth studies are needed to explore it.

A study by Kim et al. showed that D-dimer levels were significantly higher in patients with cancer-related stroke compared with patients with non-cancer-related stroke or cancer without stroke (*P* < 0.001). The study also examined ten patients with elevated D-dimer levels and stroke with multiple vascular regions for occult malignancy and found that all patients had malignant tumors. Therefore, high D-dimer levels in patients with stroke might serve as a clue to the presence of occult cancer in patients with stroke [17]. Yu et al. conducted a study to examine the clotting mechanism of colon cancer models. They found that the activation of oncogenes and/or inactivation of tumor suppressor genes upregulated the clotting activity *in vivo* (by increasing TF, PAI-1, and COX2 expressions), indicating that cancer was significantly correlated with the hypercoagulable state of patients with cancer [18]. The D-dimer levels, in this case, increased considerably, up to nine times the normal level, and there was a significant decrease in platelet aggregation function. Consequently, the tumor index increased substantially in reexamination.

Recent studies have shown that patients with cancer-related stroke exhibit similar imaging features, i.e., multi-regional cerebral infarction [17]. Previously, multiple cerebral infarctions caused by mainly unknown causes were attributed to cardiac embolism, of which atrial fibrillation was the most common. The critical correlation between “Three Territory Sign” (TTS: bilateral anterior and posterior circulation acute ischemic diffusion-weighted imaging lesions) and malignant tumors had not been fully recognized. Recent studies have indicated that TTS might be a valuable clue for the diagnosis of TS [19]. Nouh et al. conducted a retrospective analysis of patients with acute stroke undergoing MRI-DWI examination, screening 64 patients with known malignant tumors and 167 patients with atrial fibrillation. They analyzed the association between these two groups (malignant tumor and atrial fibrillation) and the number of cerebral infarctions. TTS was six times more likely to occur in the malignancy cohort than in patients with atrial fibrillation [20]. These findings suggested that TTS might be a specific marker of malignant tumor-associated cerebral infarction. Here, the head MRI of the patient showed typical signs of TTS, which was an important clue for the diagnosis of TS.

The diagnosis of TS is primarily based on imaging and coagulation tests. The possibility of TS should be considered when a patient has clinically unexplained multi-regional cerebral infarction, elevated D-dimer levels on coagulation tests, or a hypercoagulable state, such as deep vein thrombosis on vascular ultrasonography. When required, it can improve the tumor markers and ultrasound examination, as well as early detection of hidden malignant tumors and intervention.

Recanalization is still considered the most effective treatment for patients with acute ischemic stroke. Based on the current stroke treatment guidelines, patients with cancer-related stroke are not prohibited from using thrombolytic drugs during the treatment time window. Still, they may respond differently to thrombolytic medications compared with patients with stroke without cancer, requiring the need for further clinical validation. Multimodal MRI (including DWI and perfusion-weighted imaging) might be helpful in selecting whether to proceed with recirculation therapy [21]. Patients with “target dismatch pattern” (mostly dark band and small core) infarcts have been shown to have better clinical outcomes [22]. However, patients with cancer-related stroke typically show standard perfusion-weighted imaging and angiography results, with very few “target mismatch” results. Also, patients with cancer-related stroke have been known to develop progressive neurological deficits within hours to days or even weeks rather than rapidly after the onset of the disease. Thus, patients with cancer-related stroke are often not eligible for thrombolysis since they might not be in the thrombolytic time window or are unlikely to respond well to thrombolysis [23].

The treatment of cancer-related VTE is considered to be more difficult compared with non-cancer cases due to the increased recurrence rate of VTE and the risk of bleeding complications due to anticoagulation [24]. Effective prevention and treatment of VTE can reduce morbidity and mortality. Based on the latest guidelines, low molecular weight heparins (LMWH) constitute the first-line treatment for VTE and are an effective and safer means for its prevention and treatment. Where LMWH is not applicable, the ASCO 2013 VTE Prevention and Treatment Guidelines recommend the use of vitamin K antagonists, such as warfarin, with target international normalized ratios in the 2–3 range. However, the efficacy of warfarin is known to be lower than that of heparin, with a higher recurrence rate. Also, the use of direct Factor Xa inhibitors, such as rivaroxaban, Apixaban, and Edoxaban, or direct thrombin inhibitors, such as dabigatrenate, has been shown to have promising effects as a long-term alternative treatment for the prevention and treatment of VTE in high-risk cancer patients. Still, to date, there is insufficient evidence to support these novel anticoagulants [2]. Novel anticoagulants are not recommended for patients with malignant tumors and VTE, and their efficacy should be further studied before application.

In this case, the patient was administered LMWH for anticoagulation therapy, which resulted in a reduction in D-dimer levels, indicating a positive response to the treatment.

The survival time of patients with stroke in TS is approximately 4–5 months, and 25% do not survive within 30 days of the diagnosis of stroke. In the absence of an effective anti-tumor treatment, the prognosis of anticoagulant therapy alone would be worse. In this study, the patient was in the advanced stage of pancreatic cancer with multiple liver and kidney metastases, and the prognosis was poor.

## 4 Conclusion

Compared with other types of cerebral infarction, TS progresses faster and is life-threatening. A timely and accurate diagnosis is critical for a better prognosis. Patients with unexplained cerebral infarction involving multiple regions, elevated plasma D-dimer levels, and elevated cancer antigen levels should be tested for TS. For such patients, whole-body CT scanning is recommended to improve tumor marker examination, if required, to confirm the presence of latent malignant tumors.
